# Age Differences in Acute Chest Pain Care in a Multisite US Cohort

**DOI:** 10.1002/clc.70275

**Published:** 2026-04-21

**Authors:** Nicklaus P. Ashburn, Anna C. Snavely, Lyle Paukner, Michael W. Supples, Benjamin T. Hutchison, David A. Pearson, Simon A. Mahler

**Affiliations:** ^1^ Department of Emergency Medicine Wake Forest University School of Medicine Winston‐Salem North Carolina USA; ^2^ Department of Biostatistics and Data Science Wake Forest University School of Medicine Winston‐Salem North Carolina USA; ^3^ Department of Emergency Medicine Carolinas Medical Center Charlotte North Carolina USA; ^4^ Department of Epidemiology and Prevention Wake Forest University School of Medicine Winston‐Salem North Carolina USA; ^5^ Department of Implementation Science Wake Forest University School of Medicine Winston‐Salem North Carolina USA

**Keywords:** age differences, chest pain, high‐sensitivity cardiac troponin, older adults

## Abstract

**Background:**

This study aims to determine if differences in age affect safety and healthcare utilization among patients with chest pain in a multisite US ED cohort, after accounting for comorbidities and high‐sensitivity troponin (hs‐cTn).

**Methods:**

We conducted a multisite observational study using the Wake Forest Chest Pain Registry, which included patients ≥ 18 years old with chest pain across 25 EDs (01/01/2021 to 12/31/2021). Each site used an hs‐cTn chest pain protocol. Patients were categorized as older (≥ 65 years), middle‐aged (46−64 years), or young (18−45 years). The primary safety outcome was death or MI at 30 days. The primary healthcare utilization outcome was 30‐day hospitalization. Multivariable logistic regression models assessed the association between age and outcomes, adjusting for sex, race, ethnicity, obesity, smoking, rurality, coronary disease, diabetes, hypertension, hyperlipidemia, insurance, site, and hs‐cTn, with young patients as the reference.

**Results:**

Among 40 979 patients, 25.1% were older, 39.7% middle‐aged, and 35.2% young. At 30 days, death or MI occurred in 7.3% (750/10 298) of older, 3.8% (611/16 260) of middle‐aged, and 0.8% (108/14 421) of young patients. After adjustment, death or MI at 30 days was more common among older (aOR 1.57, 95% CI 1.17−2.12) and middle‐aged (aOR 1.57, 95% CI 1.22‐2.02) patients. Hospitalization at 30 days occurred in 56.3% (5799/10 298) of older, 35.4% (5761/16 260) of middle‐aged, and 12.8% (1849/14 421) of young patients. With adjustment, hospitalization remained more common among older (aOR 2.51, 95% CI 2.27−2.78) and middle‐aged (aOR 1.93, 95% CI 1.80−2.07) patients.

**Conclusion:**

After adjusting for comorbidities and hs‐cTn results, older adults had higher rates of death or MI and hospitalization at 30 days.

## Summary

- This study of > 40 000 adult patients across 25 emergency department (ED) sites aims to determine if differences in age affect safety and healthcare utilization among patients presenting to the ED with chest pain, even after accounting for comorbidities and high‐sensitivity troponin (hs‐cTn).
- After adjusting for comorbidities and hs‐cTn results, older adults had higher rates of death or MI and hospitalization at 30 days.
- These findings suggest that accelerated diagnostic protocols or algorithms for risk stratification of ED patients with chest pain should strongly consider including age as a variable.

## 1 Introduction

Each year, more than 7 million patients present to an emergency department (ED) in the US with acute chest pain [1, 2]. Older adults are at particularly high risk for acute coronary syndrome (ACS) as age is a key risk marker for coronary heart disease [3, 4, 5]. It is well known that atherosclerotic plaque burden, thromboembolic risk, multivessel coronary artery disease, and coronary calcifications increase with age [6, 7]. Increasing age is also associated with functional decline, frailty, and dementia, which complicate providing emergency care to older adults with chest pain [7, 8]. Given that older adults may present with atypical symptoms for ACS and that they account for nearly 40% of ACS admissions [5, 9, 10], it is key for emergency clinicians to appreciate the risk involved when caring for older adults presenting with acute chest pain.

Despite the increased risk and challenging nature of caring for older patients with acute chest pain, most contemporary accelerated diagnostic protocols (ADPs) are troponin‐only in nature and do not consider age or other clinical variables. For example, the European Society of Cardiology 0/1‐h algorithm, the High‐STEACS Pathway, and the American College of Cardiology's Expert Consensus Decision Pathway do not consider age when risk‐stratifying patients for ACS [11, 12, 13, 14, 15, 16]. Our team has previously shown that troponin‐only ADPs place older adults at increased risk for missed myocardial infarction (MI) compared to their middle‐aged and young counterparts [17, 18]. However, our prior studies on this topic are limited by small sample size and inclusion of a limited number of ED sites [17, 18, 19].

To better understand the role of age in adverse cardiac events and healthcare utilization, we conducted a large, prospective observational cohort study of patients presenting with acute chest pain across 25 ED sites. The primary objective was to determine and compare the rates of all‐cause death and MI as well as hospitalization at 30 days for older, middle‐aged, and young patients while adjusting for known cardiovascular disease confounders and for index visit hs‐cTnI measures. Additional objectives included examining rates of major adverse cardiovascular events (MACE: all‐cause death, MI, and revascularization) and objective cardiac testing (OCT: stress testing and coronary angiography) at 30 days across age groups. We hypothesized that older adults would have increased 30‐day cardiac events, hospitalizations, and OCT compared to young patients, even after adjusting for their comorbidities and initial high‐sensitivity cardiac troponin (hs‐cTn) measure.

## 2 Methods

### 2.1 Study Design and Oversight

We conducted a multisite observational cohort study of patients presenting to the ED with acute chest pain. Patients were prospectively accrued to the Wake Forest Chest Pain Registry from January 1, 2021, to December 31, 2021, under a waiver of informed consent. This study was approved by the institutional review board. The methods of the Wake Forest Chest Pain Registry have been previously described [13, 20, 21]. The Strengthening the Reporting of Observational Studies in Epidemiology (STROBE) guidelines helped direct the research and reporting processes [22].

### 2.2 Study Setting and Population

The Wake Forest Chest Pain Registry accrued patients from 25 EDs in North Carolina. These sites included two academic tertiary care centers and 23 community hospitals. The sites were geographically diverse and included urban, rural, and suburban areas. Across all sites, there were approximately 1 million ED encounters during the study year. Supporting Information S1: Table 1 describes each ED site. Each ED used a History, Electrocardiogram, Age, Risk factor, and Troponin (HEART) Pathway‐based ADP with high‐sensitivity cardiac troponin (hs‐cTn) for risk stratification purposes [23, 24]. Each site used the Beckman Coulter Access hs‐cTnI assay (Brea, CA), which has manufacturer reported sex‐specific 99th percentile URLs of 15 ng/L for women on the DXI platform and 12 ng/L on the Access 2 platform and 20 ng/L for men regardless of platform, with 10% coefficient of variation (CV) at 4 ng/L, and a limit of quantitation (LoQ) at 2 ng/L [25, 26, 27].

Patients ≥ 18 years old being evaluated for ACS who had a chief complaint of chest pain and at least one troponin ordered were included in the analysis. Patients with evidence of ST‐segment elevation myocardial infarction (STEMI) were excluded. Repeat visits for chest pain were identified and considered recurrent care. Patients transferred within the network were classified based on their initial ED visit, with care at the receiving hospital considered part of their index encounter. Consistent with prior cardiovascular studies, patients were classified as older (≥ 65 years), middle‐aged (46−64 years), or young (18‐45 years) [17, 18, 19].

### 2.3 Data Collection and Variables

Index encounter data (including ED, observation unit, and hospitalization data) through 30 days of follow‐up were extracted from the EHR (Cerner, Kansas City, MO and Clarity‐Epic Systems Corporation, Verona, WI). Pre‐validated, structured EHR variables and diagnosis and procedure codes (Current Procedural Technology [CPT] and International Classification of Diseases [ICD] 9 and 10 codes) were used to obtain patient demographics, comorbidities, cardiovascular risk factors, troponin results, dispositions, diagnoses, and vital status [24, 28, 29, 30, 31]. Age was determined in the EHR using the patient's self‐reported birthdate. The North Carolina State Center for Health Statistics was queried for death data not captured in our EHR. As with prior EHR‐based cardiovascular cohort studies, we assumed complete follow‐up [13, 17, 20, 24, 32, 33]. We previously found that these methods missed very few outcome events [24, 34].

### 2.4 Outcomes

The primary safety outcome was the composite of all‐cause death or MI at 30 days, inclusive of the index encounter. Secondary safety outcomes included major adverse cardiovascular events (MACE: the composite of all‐cause death, MI, and coronary revascularization) at 30 days as well as the individual MACE subcomponents at index through 30 days. Coronary revascularization included any percutaneous coronary intervention (PCI) with or without stent placement and coronary artery bypass grafting (CABG).

The primary healthcare utilization outcome was hospitalization at 30 days, which included inpatient and observation unit admissions, regardless of cause. Secondary healthcare utilization outcomes included OCT at 30 days, which was defined as non‐invasive testing (NIT: stress testing and coronary computed tomography angiography [CCTA]) and invasive coronary angiography.

### 2.5 Statistical Analysis

Frequencies and percentages or medians and interquartile ranges (IQRs) were used to describe the characteristics of patients within each age group. To assess the association between age group and each outcome, multivariable logistic regression was performed. Models were adjusted for sex, race, ethnicity, obesity, smoking, rurality, coronary artery disease, diabetes, hypertension, hyperlipidemia, insurance status, ED site, and initial hs‐cTn. Rurality was determined as either urban or rural using the Federal Office of Rural Health Policy definition [35]. Hs‐cTn was treated as a binary variable using the sex‐specific 99th percentile URL hs‐cTnI cut‐points. These variables were selected *a priori* due to their relevance and inclusion in previous cardiovascular risk stratification studies [13, 15, 17, 19, 20, 21]. Missing obesity and smoking status were imputed by predictive mean matching using all predictors and outcome variables to create 10 data sets with complete obesity and smoking status data. No other covariates required imputation. Separate, unadjusted logistic models were also fit to evaluate the association between age and each outcome. Young patients were considered the reference group for the primary analyses. Unadjusted odds ratios (ORs) or adjusted ORs (aORs) with corresponding 95% confidence intervals (95% CI) were calculated as appropriate for each model. We conducted two additional pre‐specified analyses. In the first, we assessed the association between age group and the study outcomes using the methods above, except that we compared older patients to middle‐aged patients. In the second analysis, we examined safety outcomes and healthcare utilization event rates by decade of life (e.g., 18−29 years, 30−39 years, 40−49 years, etc.).

## 3 Results

**Figure 1:**
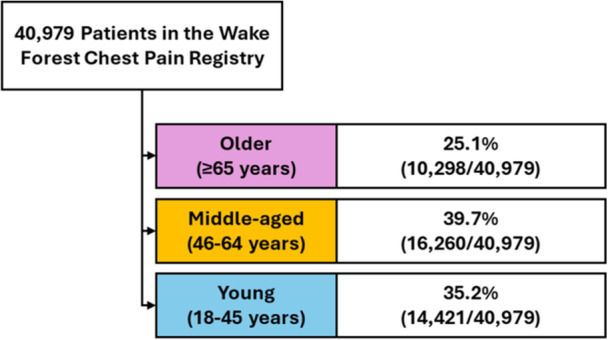
Study flow diagram.

During the 12‐month study period, 40 979 patients were evaluated for acute chest pain. The cohort was 56.6% (23 188/40 979) female, 41.3% non‐White, (16 929/40 979), and had a median age of 52 (IQR 40−65) years. Older patients accounted for 25.1% (10 298/40 979), middle‐aged patients 39.7% (16 260/40 979), and young patients 35.2% (14 421/40 979) of the sample. At 30 days, the primary safety outcome of death or MI occurred in 3.6% (1469/40 979) and MACE in 4.5% (1828/40 979) of the overall cohort. Among all patients, 32.7% (13409/40 979) were hospitalized and 16.9% (6940/40 979) received OCT at 30 days. Table 1 describes the cohort. Figure 1 shows the study flow diagram.

**Table 1:** Description of the cohort.

Characteristic	Older	Middle‐aged	Young
	(≥ 65 years)	(46–64 years)	(18−45 years)
	*n* = 10 298	*n* = 16 260	*n* = 14 421
	*n* (%)	*n* (%)	*n* (%)
Age, median (IQR), years	74 (69‐79)	54 (50‐59)	35 (29‐41)
Female	5838 (56.7)	8951 (55.0)	8399 (58.2)
*Race/Ethnicity*			
Black, Not Hispanic	2354 (22.9)	5620 (34.6)	5506 (38.2)
Hispanic or Latino	276 (2.7)	755 (4.6)	1164 (8.1)
Other	232 (2.2)	495 (3.0)	514 (3.6)
White, Not Hispanic	7436 (72.2)	9390 (57.8)	7237 (50.2)
Insurance status			
Other	358 (3.5)	3104 (19.1)	4007 (27.8)
Private	702 (6.8)	8065 (49.6)	6558 (45.5)
Public	9238 (89.7)	5091 (31.3)	3856 (26.7)
Rurality			
Rural	2651 (25.7)	3741 (23.0)	2798 (19.4)
Urban	7647 (74.3)	12519 (77.0)	11 623 (80.6)
*Risk factors*			
BMI ≥ 30 kg/m^2*^	5705 (54.1)	7175 (55.7)	3456 (40.8)
Smoking^±^	4191 (40.8)	6789 (42.0)	4757 (33.4)
Diabetes	2502 (24.3)	2625 (16.1)	654 (4.5)
Coronary artery disease	2897 (28.1)	2050 (12.6)	186 (1.3)
Hypertension	7299 (70.9)	8175 (50.3)	2675 (18.6)
Hyperlipidemia	6910 (67.1)	7379 (45.4)	2132 (14.8)
Elevated Initial hs‐cTn	2254 (21.9)	1785 (11.0)	610 (4.2)

*Note:* *1823 older, 3375 middle‐aged, and 3883 young patients were missing BMI; ±25 older, 97 middle‐aged, and 166 young patients were missing smoking status.

Abbreviations: BMI, body mass index; hs‐cTn, high‐sensitivity cardiac troponin; IQR, interquartile range.

The primary safety outcome of death or MI at 30 days occurred in 7.3% (750/10 298) of older, 3.8% (611/16 260) of middle‐aged, and 0.8% (108/14 421) of young patients. At 30 days, MACE occurred in 9.2% (943/10 298) of older, 4.7% (765/16 260) of middle‐aged, and 0.8% (120/14 421) of young patients. After adjusting for comorbidities and hs‐cTn results, older adults still had higher rates of 30‐day death or MI (aOR 1.57, 95% CI 1.17−2.12) and 30‐day MACE (aOR 1.79, 95% CI 1.36−2.36) than young patients. Similarly, 30‐day death or MI (aOR 1.57, 95% CI 1.22−2.02) and 30‐day MACE (aOR 1.63, 95% CI 1.29−2.06) rates were higher among middle‐aged patients than young patients. Table 2 describes the safety events by age group. Table 3 presents the unadjusted and aORs for safety events at index through 30 days.

**Table 2:** Safety and healthcare utilization outcomes from index through 30 days by age group.

	Older	Middle‐aged	Young
	(≥ 65 years)	(46−64 years)	(18−45 years)
	*n* = 10298	*n* = 16 260	*n* = 14 421
	*n* (%)	*n* (%)	*n* (%)
Safety			
*Index*			
MI	632 (6.1)	511 (3.1)	83 (0.6)
Death	75 (0.7)	37 (0.2)	7 (0.1)
Revascularization	445 (4.3)	391 (2.4)	29 (0.2)
Death or MI	645 (6.3)	521 (3.2)	88 (0.6)
MACE	805 (7.8)	656 (4.0)	94 (0.7)
*30‐day follow‐up*			
MI	224 (2.2)	162 (1.0)	29 (0.2)
Death	163 (1.6)	66 (0.4)	9 (0.1)
Revascularization	90 (0.9)	68 (0.4)	13 (0.1)
Death or MI	267 (2.6)	184 (1.1)	35 (0.2)
MACE	320 (3.1)	219 (1.4)	42 (0.3)
*30 days (index + follow‐up)*			
MI	702 (6.8)	580 (3.6)	97 (0.7)
Death	198 (1.9)	88 (0.5)	15 (0.1)
Revascularization	523 (5.1)	453 (2.8)	40 (0.3)
Death or MI	750 (7.3)	611 (3.8)	108 (0.8)
MACE	943 (9.2)	765 (4.7)	120 (0.8)
Healthcare utilization			
*Index*			
Hospitalization	5445 (52.9)	5257 (32.3)	1492 (10.4)
Objective cardiac testing	2552 (24.8)	2961 (18.2)	499 (3.5)
Non‐invasive testing	1710 (16.6)	2244 (13.8)	415 (2.9)
Invasive coronary angiography	915 (8.9)	780 (4.8)	94 (0.7)
*Follow‐up*			
Hospitalization	953 (9.6)	943 (5.8)	498 (3.5)
Objective cardiac testing	431 (4.2)	532 (3.3)	190 (1.3)
Non‐invasive testing	377 (3.7)	494 (3.0)	180 (1.3)
Invasive coronary angiography	201 (2.0)	197 (1.2)	43 (0.3)
*30 days (index + follow‐up)*			
Hospitalization	5799 (56.3)	5761 (35.4)	1849 (12.8)
Objective cardiac testing	2887 (28.0)	3387 (20.8)	666 (4.6)
Non‐invasive testing	2018 (19.6)	2,652 (16.3)	576 (4.0)
Invasive coronary angiography	964 (9.4)	815 (5.0)	103 (0.7)

Abbreviations: MACE, major adverse cardiovascular event (death, MI, or revascularization); MI, myocardial infraction.

**Table 3:** Unadjusted and adjusted odds ratios for index and 30‐day study outcomes for older and middle‐aged patients, with young patients being the reference group.

	Older: Young		Middle‐aged: Young	
Outcome	OR (95% CI)	aOR (95% CI)	OR (95% CI)	aOR (95% CI)
Safety				
*Index*				
MI	11.29 (8.97, 14.22)	1.44 (1.03, 2.02)	5.61 (4.44, 7.08)	1.55 (1.16, 2.07)
Death or MI	10.88 (8.7, 13.62)	1.40 (1.01, 1.95)	5.39 (4.3, 6.77)	1.52 (1.15, 2.01)
MACE	12.92 (10.42, 16.03)	1.62 (1.19, 2.21)	6.41 (5.16, 7.96)	1.62 (1.24, 2.12)
*30 days (index + follow‐up)*				
MI	10.80 (8.72, 13.38)	1.52 (1.11, 2.08)	5.46 (4.4, 6.78)	1.56 (1.20,2.03)
Death or MI	10.41 (8.49, 12.76)	1.57 (1.17, 2.12)	5.17 (4.21, 6.36)	1.57 (1.22, 2.02)
MACE	12.01 (9.92, 14.55)	1.79 (1.36, 2.36)	5.88 (4.85, 7.14)	1.63 (1.29, 2.06)
Healthcare utilization				
*Index*				
Hospitalization	9.72 (9.1, 10.39)	2.66 (2.39, 2.96)	4.14 (3.89, 4.41)	2.03 (1.88, 2.19)
Objective cardiac testing	9.19 (8.32, 10.16)	3.07 (2.64, 3.57)	6.21 (5.63, 6.85)	2.88 (2.58, 3.21)
Non‐invasive testing	6.72 (6.02, 7.51)	3.46 (2.93, 4.07)	7.68 (6.19, 9.52)	1.82 (1.42, 2.35)
Invasive coronary angiography	14.86 (12.0, 18.41)	1.71 (1.27, 2.32)	5.40 (4.85, 6.02)	3.00 (2.67, 3.38)
*30 Days (Index + Follow‐up)*				
Hospitalization	8.76 (8.23, 9.33)	2.51 (2.27, 2.78)	3.73 (3.52, 3.96)	1.93 (1.8, 2.07)
Objective cardiac testing	8.05 (7.36, 8.79)	3.03 (2.64, 3.47)	5.43 (4.98, 5.92)	2.68 (2.43, 2.95)
Non‐invasive testing	5.86 (5.32, 6.45)	3.35 (2.89, 3.87)	4.68 (4.27, 5.14)	2.77 (2.5, 3.07)
Invasive coronary angiography	14.36 (11.7, 17.62)	1.73 (1.29, 2.31)	7.34 (5.97, 9.02)	1.77 (1.38, 2.25)

Abbreviations: aOR, adjusted odds ratio; CI, confidence interval, MACE, major adverse cardiovascular event (death, MI, or revascularization); OR, odds ratio.

The primary healthcare utilization outcome of hospitalization at 30 days occurred in 56.3% (5799/10 298) of older, 35.4% (5761/16 260) of middle‐aged, and 12.8% (1849/14 421) of young patients. OCT at 30 days was obtained in 28.0% (2887/10 298) of older, 20.8% (3387/16 260) of middle‐aged, and 4.6% (666/14 421) of young patients. With adjustment, hospitalization at 30 days remained more common among older (aOR 2.51, 95% CI 2.27−2.78) and middle‐aged (aOR 1.93, 95% CI: 1.80−2.07) patients than young patients. OCT was also more common among older (aOR 3.03, 95% CI 2.64−3.47) and middle‐aged (aOR 2.68, 95% CI 2.43−2.95) patients compared to young patients. Table 2 describes healthcare utilization by age group. Table 3 shows the unadjusted and aORs for healthcare utilization outcomes at index through 30 days. Supporting Information S1: Table 2 presents healthcare utilization outcomes by decade of life.

In our pre‐specified analysis evaluating the association between age with the study outcomes, where middle‐aged patients were the reference group, we found that older adults had similar rates of 30‐day death or MI (aOR 0.99, 95% CI 0.84−1.15) and 30‐day MACE (aOR 1.05, 95% CI 0.91−1.21). However, older adults had increased hospitalizations (aOR 1.36, 95% CI 1.26−1.47) and OCT (aOR 1.17, 95% CI 1.08−1.27) compared to middle‐aged patients. When examining outcomes by each decade of life, we found that 30‐day safety events and healthcare utilization tended to become more prevalent with each decade of life. However, OCT rates trended down among the oldest adults (≥ 90 years). Supporting Information S1: Table 2 presents study outcomes by decade of life. Supporting Information S1: Table 3 presents the association between age group with study outcome among older adults with middle‐aged adults as the reference group.

## 4 Discussion

In this large multisite U.S. ED cohort study, we found that older and middle‐aged patients were more likely to have death or MI at 30 days compared to young patients after adjustment for key comorbidities associated with cardiovascular disease and index visit hs‐cTn measures. We also observed higher adjusted odds of healthcare utilization among older and middle‐aged adults for hospitalization and OCT. Thus, this study provides further evidence that age remains a key independent cardiovascular risk factor for ED patients with acute chest pain, even in the era of hs‐cTn. This suggests that future ADPs should incorporate age when risk‐stratifying patients for possible ACS and that many existing hs‐cTn ADPs, which do not include age as a variable, may underestimate risk in older patients.

While adjusting for well‐known cardiovascular comorbidities and hs‐cTn results did attenuate the effect size, age remained strongly associated with death, MI, and MACE at 30‐days. It is well known that increasing age is associated with greater atherosclerotic plaque burden, thromboembolic risk, and multivessel coronary calcifications, leading to increased cardiovascular events [6, 7]. However, our findings suggest that hs‐cTn and other cardiovascular risk factor phenotypes (e.g., hyperlipidemia, diabetes, etc.) insufficiently explain this disease and risk progression. While hs‐cTn is the gold‐standard biomarker for myocardial injury and does have important prognostic value for downstream cardiovascular risk, it is not a marker of atherosclerosis burden or even myocardial ischemia in the absence of infarction. Thus, unless novel biomarkers or clinical variables are uncovered that more fully explain risk progression with aging, incorporating patient age into future ADPs may be necessary for accurate risk stratification. Given the widespread use of troponin‐only ADPs, which risk‐stratify patients solely based on hs‐cTn measures, these findings are highly clinically relevant. Further, these data are consistent with prior data demonstrating that troponin‐only ADPs, such as the European Society of Cardiology 0/1‐h algorithm, have high rates of missed cardiac events among older and middle‐aged patients in the United States [19].

In addition to demonstrating an increased adjusted odds of cardiac events with increasing age, our study also demonstrated that healthcare resource use, in the form of hospitalizations and OCT, increased with age after adjusting for comorbidities and hs‐cTn measures. These findings were not consistent with prior literature, as numerous prior studies have reported cardiovascular care disparities and under‐testing among older adults [7, 10, 36]. In fact, it seems likely that older adults were actually being over‐tested in our cohort. For example, 9.4% of older adults had invasive coronary angiography, but just 5.1% had a revascularization procedure performed at 30 days. In light of the ISCHEMIA and COURAGE trials and the potential for over‐testing among older adults, clinicians should thoughtfully consider the role of invasive angiography with potential PCI compared to medical management alone [37, 38, 39].

We also found a low prevalence of cardiac events among young patients in this cohort, with just 0.8% having 30‐day death or MI. Despite < 1% of young patients having an adverse event, more than 10% were hospitalized, and nearly 5% received OCT, further suggesting that clinicians are likely over‐testing in this age group. In contrast to the low prevalence of disease among young patients in this cohort, other chest pain cohorts report that 2%–7% of young patients have death or MI at 30 days [17, 18, 19, 40, 41]. Therefore, even though we found young patients to have a very low event rate in this cohort, clinicians must consider the pre‐test probability of disease in their patient population before determining that hospitalization and OCT are not indicated in young patients.

Future studies focused on cardiovascular care differences driven by age should aim to elucidate the mechanism driving the increased risk conferred by age. Ideally, future chest pain risk stratification work will account for frailty, which we are unable to adjust for in our current chest pain registry. Additionally, while we adjusted for initial hs‐cTnI using the established sex‐specific 99th percentile URL cut‐points, age‐specific hs‐cTn cut‐points are not presently recommended [42, 43]. It is unknown whether age‐specific hs‐cTn cut‐points would improve chest pain risk stratification, particularly among older adults. Finally, in the future, machine learning and artificial intelligence risk stratification tools, such as ARTEMIS and CoDE‐ACS, will likely leverage a large number of clinical variables, including age [44, 45]. We believe this will be important, especially as this study adds to a growing body of literature showing that age is a key risk marker, even after accounting for comorbidities and hs‐cTn.

## 5 Limitations

This study has limitations. Although this study was conducted in one state, we accrued patients from 25 diverse EDs that include rural, suburban, and urban areas. The EDs also include both academic tertiary care centers and community hospitals. While our use of the EHR to identify outcome events may be less reliable than traditional follow‐up methods, it is pragmatic and allowed us to evaluate more than 40 000 ED patients. Additionally, our prior studies suggest a high degree of validity with using the EHR for follow‐up, as we have traditionally missed very few events [13, 20, 24]. It is important to note that our outcomes are not adjudicated, thus risking misclassification bias. Furthermore, we are also unable to determine if death and hospitalization events were cardiac‐related or not.

## 6 Conclusion

In this large multisite US study, we observed that older and middle‐aged patients presenting to the ED with acute chest pain were more likely to have death or MI at 30 days than young patients, even after adjustment for comorbidities and hs‐cTn measures. Older and middle‐aged patients were also hospitalized more frequently and received more cardiac testing than young patients. While clinicians might expect older patients to have higher rates of death or MI and healthcare utilization due to their comorbidities, we found that older and middle‐aged patients were still at higher risk of adverse events and healthcare utilization even after adjusting for comorbidities and hs‐cTn. These findings suggest that ADPs or algorithms for risk stratification of ED patients with possible ACS should strongly consider including age as a variable.

## Author Contributions

Nicklaus P. Ashburn, Simon A. Mahler, and Anna C. Snavely conceived the study idea. Lyle Paukner and Anna C. Snavely coordinated data management. Anna C. Snavely and Lyle Paukner performed the data analysis. David A. Pearson assisted with trial coordination. All authors contributed to the manuscript and its revision. Nicklaus P. Ashburn takes responsibility for the manuscript as a whole.

## Conflicts of Interest

Dr. Ashburn receives funding from NHLBI (K23HL169929), AHRQ (R01HS029017), and the Emergency Medicine Foundation.

Dr. Snavely receives funding from NHLBI (K23HL169929), Abbott Laboratories, HRSA (1H2ARH399760100), AHRQ (R01HS029017 and R21HS029234), and the Emergency Medicine Foundation.

Dr. Supples receives funding from the NIH (UL1TR001420), AHRQ (R01HS029017), and the National Foundation of Emergency Medicine.

Dr. Hutchison receives funding from the Emergency Medicine Foundation.

Dr. Pearson is an investor and advisor for Power Medical Inc.

Dr. Mahler receives funding/support from Roche Diagnostics, Abbott Laboratories, QuidelOrtho, Siemens, Grifols, Polymedco, Beckman Coulter, Genetesis, Cytovale, National Foundation of Emergency Medicine, BlueJay Diagnostics, Reprieve Cardiovascular, Duke Endowment, Brainbox, HRSA (1H2ARH399760100), the Emergency Medicine Foundation, and AHRQ (R01HS029017 and R21HS029234). He is a consultant for Roche, QuidelOrtho, Abbott, Siemens, Polymedco, Inflammatix, and Radiometer, and is the Chief Medical Officer for Impathiq Inc. The other authors declare no conflicts of interest.

## Supporting information

Supplemental Material ‐ Age ‐ CC.

Supplementary Information

## Data Availability

The data, analytic methods, and study materials will not be made available to other researchers.
