# Supplementary material for: Age Differences in Acute Chest Pain Care in a Multisite US Cohort
Source: Clin Cardiol. 2026 Apr 21;49(4):e70275. doi: 10.1002/clc.70275 (PMC13098536; doi:10.1002/clc.70275)
Supplement: Supplementary file 1 — Supplemental Material ‐ Age ‐ CC. [file CLC-49-e70275-s002.docx]

**SUPPLEMENTAL MATERIAL**

**Table S1.** ED site descriptions and volume by age group.

| **Site** | **Hospital Name** | **City** | **State** | **Type** | **Setting** | **Annual ED Volume** | **Accruement by Age Group per Site** | | |
| --- | --- | --- | --- | --- | --- | --- | --- | --- | --- |
|  |  |  |  |  |  |  | **Older**  **(≥65** **years)**  n = 10298  n (%) | **Middle-aged**  **(46-64** **years)** n = 16260  n (%) | **Young**  **(18-45** **years)**  n = 14421  n (%) |
| 1 | Atrium Health Anson | Wadesboro | NC | Community | Rural | 10,000 | 135 (1.3) | 236 (1.4) | 220 (1.5) |
| 2 | Atrium Health Ballantyne | Charlotte | NC | Community | Urban | 15,000 | 341 (3.3) | 495 (3.0) | 331 (2.3) |
| 3 | Atrium Health Cabarrus | Concord | NC | Community | Suburban | 61,000 | 1103 (10.7) | 1314 (8.1) | 1080 (7.5) |
| 4 | Atrium Health Cleveland | Shelby | NC | Community | Suburban | 66,000 | 664 (6.5) | 967 (6.0) | 612 (4.2) |
| 5 | Atrium Health Harrisburg | Harrisburg | NC | Community | Suburban | 20,000 | 184 (1.8) | 457 (2.8) | 533 (3.7) |
| 6 | Atrium Health Huntersville | Huntersville | NC | Community | Suburban | 25,000 | 141 (1.4) | 332 (2.0) | 387 (2.7) |
| 7 | Atrium Health Kannapolis | Kannapolis | NC | Community | Suburban | 18,000 | 249 (2.4) | 493 (3.0) | 565 (3.9) |
| 8 | Atrium Health Kings Mountain | Kings Mountain | NC | Community | Rural | 32,000 | 87 (0.8) | 226 (1.4) | 289 (2.0) |
| 9 | Atrium Health Lincoln | Lincolnton | NC | Community | Suburban | 45,000 | 467 (4.5) | 732 (4.5) | 523 (3.6) |
| 10 | Atrium Health Mercy | Charlotte | NC | Community | Urban | 36,000 | 256 (2.5) | 587 (3.6) | 563 (3.9) |
| 11 | Atrium Health Pineville | Charlotte | NC | Community | Urban | 60,000 | 1016 (9.9) | 1158 (7.1) | 967 (6.7) |
| 12 | Atrium Health Providence | Charlotte | NC | Community | Urban | 14,000 | 119 (1.2) | 286 (1.8) | 268 (1.9) |
| 13 | Atrium Health SouthPark | Charlotte | NC | Community | Urban | 13,000 | 160 (1.6) | 295 (1.8) | 415 (2.9) |
| 14 | Atrium Health Stanly | Albemarle | NC | Community | Rural | 33,000 | 403 (3.9) | 495 (3.0) | 302 (2.1) |
| 15 | Atrium Health Steele Creek | Charlotte | NC | Community | Urban | 16,000 | 272 (2.6) | 673 (4.1) | 880 (6.1) |
| 16 | Atrium Health Union | Monroe | NC | Community | Suburban | 60,000 | 464 (4.5) | 757 (4.7) | 603 (4.2) |
| 17 | Atrium Health University City | Charlotte | NC | Community | Urban | 81,000 | 443 (4.3) | 1078 (6.6) | 1252 (8.7) |
| 18 | Atrium Health Waxhaw ED | Waxhaw | NC | Community | Suburban | 17,000 | 196 (1.9) | 308 (1.9) | 254 (1.8) |
| 19 | Blue Ridge Valdese | Valdese | NC | Community | Rural | 19,000 | 95 (0.9) | 202 (1.2) | 180 (1.2) |
| 20 | Carolinas Medical Center | Charlotte | NC | Tertiary Care Center | Urban | 115,000 | 675 (6.5) | 1116 (6.9) | 875 (6.1) |
| 21 | Davie Medical Center | Bermuda Run | NC | Community | Suburban | 22,000 | 265 (2.6) | 458 (2.8) | 402 (2.8) |
| 22 | High Point Medical Center | High Point | NC | Community | Urban | 60,000 | 907 (8.8) | 1164 (7.2) | 886 (6.1) |
| 23 | Lexington Medical Center | Lexington | NC | Community | Suburban | 35,000 | 339 (3.3) | 498 (3.1) | 469 (3.2) |
| 24 | Wake Forest Baptist Medical Center | Winston-Salem | NC | Tertiary Care Center | Urban | 114,000 | 942 (9.2) | 1466 (9.0) | 1165 (8.1) |
| 25 | Wilkes Medical Center | Wilkesboro | NC | Community | Rural | 33,000 | 375 (3.6) | 467 (2.9) | 400 (2.8) |

ED – emergency department, NC – North Carolina

**Table S2.** Outcomes by each decade of life.

|  | **18-29** **years** n = 4,090  n (%) | **30-39** **years** n = 6,255  n (%) | **40-49 years**  n = 7,991  n (%) | **50-59** **years** n = 8,715  n (%) | **60-69 years**  n = 6,649  n (%) | **70-79** **years** n = 4,861  n (%) | **80-89** **years** n = 2,036  n (%) | **≥90** **years** n = 382  n (%) |
| --- | --- | --- | --- | --- | --- | --- | --- | --- |
| **SAFETY** |  |  |  |  |  |  |  |  |
| **Index** |  |  |  |  |  |  |  |  |
| MI | 6 (0.1) | 32 (0.5) | 114 (1.4) | 272 (3.1) | 358 (5.4) | 303 (6.2) | 118 (5.8) | 23 (6.0) |
| Death | 0 (0.0) | 4 (0.1) | 6 (0.1) | 20 (0.2) | 27 (0.4) | 39 (0.8) | 16 (0.8) | 7 (1.8) |
| Revascularization | 1 (0.0) | 8 (0.1) | 68 (0.8) | 197 (2.3) | 289 (4.3) | 239 (4.9) | 59 (2.9) | 4 (1.1) |
| Death or MI | 6 (0.1) | 34 (0.5) | 117 (1.5) | 276 (3.2) | 367 (5.5) | 308 (6.3) | 121 (5.9) | 25 (6.5) |
| MACE | 6 (0.1) | 36 (0.6) | 140 (1.8) | 337 (3.9) | 470 (7.1) | 399 (8.2) | 140 (6.9) | 27 (7.1) |
| **30-day Follow-up** |  |  |  |  |  |  |  |  |
| MI | 2 (0.1) | 13 (0.2) | 34 (0.4) | 97 (1.1) | 109 (1.6) | 105 (2.2) | 48 (2.4) | 7 (1.8) |
| Death | 1 (0.0) | 5 (0.1) | 13 (0.2) | 27 (0.3) | 59 (0.9) | 76 (1.6) | 39 (1.9) | 18 (4.7) |
| Revascularization | 1 (0.0) | 4 (0.1) | 11 (0.1) | 41 (0.5) | 56 (0.8) | 44 (0.9) | 13 (0.6) | 1 (0.3) |
| Death or MI | 3 (0.1) | 15 (0.2) | 40 (0.5) | 106 (1.2) | 128 (1.9) | 123 (2.5) | 58 (2.9) | 13 (3.4) |
| MACE | 4 (0.1) | 18 (0.3) | 44 (0.6) | 127 (1.5) | 158 (2.4) | 152 (3.1) | 65 (3.2) | 13 (3.4) |
| **30 Days (Index + Follow-up)** |  |  |  |  |  |  |  |  |
| MI | 7 (0.2) | 37 (0.6) | 132 (1.6) | 312 (3.6) | 397 (6.0) | 330 (6.8) | 137 (6.7) | 27 (7.1) |
| Death | 1 (0.0) | 8 (0.1) | 18 (0.2) | 38 (0.4) | 75 (1.1) | 92 (1.9) | 48 (2.4) | 21 (5.5) |
| Revascularization | 1 (0.0) | 12 (0.2) | 78 (1.0) | 235 (2.7) | 338 (5.1) | 277 (5.7) | 70 (3.4) | 5 (1.3) |
| Death or MI | 8 (0.2) | 41 (0.7) | 141 (1.8) | 325 (3.7) | 424 (6.4) | 349 (7.2) | 148 (7.3) | 33 (8.6) |
| MACE | 8 (0.2) | 46 (0.7) | 166 (2.1) | 399 (4.6) | 543 (8.2) | 461 (9.5) | 170 (8.3) | 35 (9.2) |
| **HEALTHCARE UTILIZATION** |  |  |  |  |  |  |  |  |
| **Index** |  |  |  |  |  |  |  |  |
| Hospitalization | 251 (6.1) | 610 (9.8) | 1,566 (19.6) | 2,781 (31.9) | 2,984 (44.9) | 2,615 (53.8) | 1,166 (57.3) | 221 (57.9) |
| Objective Cardiac Testing | 33 (0.8) | 175 (2.8) | 827 (10.3) | 1,581 (18.1) | 1,619 (24.4) | 1,308 (26.9) | 431 (21.2) | 38 (9.9) |
| Non-invasive Testing | 24 (0.6) | 146 (2.3) | 686 (8.6) | 1,217 (14.0) | 1,104 (16.6) | 859 (17.7) | 302 (14.8) | 31 (8.1) |
| Invasive Coronary Angiography | 10 (0.2) | 30 (0.5) | 160 (2.0) | 395 (4.5) | 558 (8.4) | 489 (10.1) | 139 (6.8) | 8 (2.1) |
| **30-day Follow-up** |  |  |  |  |  |  |  |  |
| Hospitalization | 113 (2.8) | 209 (3.3) | 342 (4.3) | 494 (5.7) | 534 (8.0) | 444 (9.1) | 215 (10.6) | 43 (11.3) |
| Objective Cardiac Testing | 22 (0.5) | 66 (1.1) | 220 (2.8) | 264 (3.0) | 283 (4.3) | 209 (4.3) | 83 (4.1) | 6 (1.6) |
| Non-invasive Testing | 22 (0.5) | 63 (1.0) | 210 (2.6) | 244 (2.8) | 249 (3.7) | 185 (3.8) | 73 (3.6) | 5 (1.3) |
| Invasive Coronary Angiography | 2 (0.1) | 11 (0.2) | 54 (0.7) | 107 (1.2) | 129 (1.9) | 109 (2.2) | 26 (1.3) | 3 (0.8) |
| **30 Days (Index + Follow-up)** |  |  |  |  |  |  |  |  |
| Hospitalization | 335 (8.2) | 766 (12.2) | 1,783 (22.3) | 3,038 (34.9) | 3,234 (48.6) | 2,781 (57.2) | 1,239 (60.9) | 233 (61.0) |
| Objective Cardiac Testing | 54 (1.3) | 235 (3.8) | 1,008 (12.6) | 1,793 (20.6) | 1,847 (27.8) | 1,473 (30.3) | 490 (24.1) | 40 (10.5) |
| Non-invasive Testing | 45 (1.1) | 204 (3.3) | 861 (10.8) | 1,422 (16.3) | 1,311 (19.7) | 1,011 (20.8) | 358 (17.6) | 34 (8.9) |
| Invasive Coronary Angiography | 10 (0.2) | 33 (0.5) | 169 (2.1) | 416 (4.8) | 587 (8.8) | 513 (10.6) | 146 (7.2) | 8 (2.1) |

**Table S3.** Unadjusted and adjusted odds ratios for index and 30-day study outcomes for older patients, with middle-aged patients as the reference group.

|  | **Older:Middle-aged** | |
| --- | --- | --- |
| **Outcome** | **OR (95%CI)** | **aOR (95%CI)** |
| **SAFETY** |  |  |
| **Index** |  |  |
| MI | 2.02  (1.79,2.27) | 0.96  (0.81,1.15) |
| Death or MI | 2.02  (1.79, 2.27) | 0.97  (0.81, 1.15) |
| MACE | 2.02  (1.81, 2.24) | 1.00  (0.85, 1.16) |
| **30 Days (Index + Follow-up)** |  |  |
| MI | 1.98  (1.77,2.21) | 0.96  (0.81,1.13) |
| Death or MI | 2.01  (1.8, 2.25) | 0.99  (0.84, 1.15) |
| MACE | 2.04  (1.85, 2.25) | 1.05  (0.91,1.21) |
| **HEALTHCARE UTILIZATION** |  |  |
| **Index** |  |  |
| Hospitalization | 2.35  (2.23, 2.47) | 1.42  (1.31, 1.53) |
| Objective Cardiac Testing | 1.48  (1.39, 1.57) | 1.13  (1.04, 1.24) |
| Non-invasive Testing | 1.24  (1.16, 1.33) | 1.18  (1.07, 1.29) |
| Invasive Coronary Angiography | 1.94  (1.75, 2.14) | 1.03  (0.89, 1.19) |
| **30 Days (Index + Follow-up)** |  |  |
| Hospitalization | 2.35  (2.23, 2.47) | 1.36  (1.26, 1.47) |
| Objective Cardiac Testing | 1.48  (1.4, 1.57) | 1.17  (1.08, 1.27) |
| Non-invasive Testing | 1.25  (1.17, 1.33) | 1.21  (1.11, 1.32) |
| Invasive Coronary Angiography | 1.96  (1.78, 2.16) | 1.05  (0.91, 1.21) |

OR – odds ratio, aOR – adjusted odds ratio, CI – confidence interval, MACE – major adverse cardiovascular event (death, MI, or revascularization)
